# Transactions of the Buffalo Obstetrical Society

**Published:** 1890-04

**Authors:** William C. Krauss

**Affiliations:** Secretary, Editor of the Transactions


### TRANSACTIONS OF THE BUFFALO OBSTETRICAL SOCIETY.

Reported by WILLIAM C. KRAUSS, M.D.., Secretary, Editor of the Transactions.

STATED meeting, February 25, 1890, held at 273 Franklin street. The President, Dr. C. C. FREDERICK, in the chair.

Dr. DELANCEY ROCHESTER read the paper of the evening, entitled,

### TWO CASES OF GASTRO-INTESTINAL DISTURBANCE COMPLICATING THE LATTER MONTHS OF PREGNANCY.

All of the works on obstetrics make mention of the gastro-intestinal disturbances of the first trimester of pregnancy, but few make any reference, more than mere mention, of such disturbances in the latter months of gestation.

I wish to call the attention of the Society this evening to two cases. I present only two cases because those that responded to ordinary hygienic measures I do not consider as worth reporting.

The first case is as follows:

CASE I.—On the 7th of April, 1887, I was summoned in haste to see a woman who expected to be confined some two months later. On my arrival at the house I found the patient lying on the bed, writhing in pain. She was the mother of several children, and, in reply to a question, said that the pain was entirely different from the pains of child-birth, except in its intermittent character. I made a bimanual examination during one of the paroxysms, and became convinced that the pains were not produced by uterine contractions, and that I must look elsewhere for the cause. The pain was in the abdomen, more especially located in the epigastrium and left hypochondrium at first, but soon spreading over the whole abdomen. I immediately asked after the state of the bowels, and was informed that they moved regularly every day, and had moved that morning. I satisfied myself that the bladder was empty, and, by palpation of the upper part of the abdomen, a tumor was discovered on the left side, extending into the lumbar region below, and apparently continuous with the spleen in the hypochondriac region above. The tumor itself was only slightly sensitive, but pressure upon it produced a recurrence of the pain, on account of which I had been called. The pain was so intense as to require pretty good-sized doses of morphine by the mouth to quiet it. I did not resort to hypodermic injection for fear of injury to the fetus in utero.

I did not manipulate the tumor extensively that day on account of the great pain produced by touching the part. Examination on the next day proved the tumor to be about four inches wide, extending about six inches below the border of the ribs, and continuous, as far as percussion could show, with the spleen above. It was slightly movable, and apparently a little more yielding than an ordinary spleen, but not so soft that it could be in the least indented, even upon hard pressure. Moreover, hard pressure always produced severe pain. I could not rid myself of the idea that it was impacted feces, although the patient insisted that she had regularly had a free daily movement of the bowels. Nevertheless I ordered a rectal enema to be given, high up in the bowel, of one pint of a saturated solution of epsom salts. This produced a tremendous discharge of fecal matter, and the tumor and all its unpleasant symptoms disappeared. I told her to make occasional use of salts through the rest of her pregnancy, and everything went on well. Constipation in the latter months of pregnancy is of very common occurrence, but it seldom goes on to the formation and retention of such large scybalous masses as in this case.

The peculiarity of this case is that the accumulation took place in spite of the fact that the patient had a daily evacuation from the bowels of a quantity which she considered sufficient.

In the treatment of the obstinate constipation of pregnancy I have found nothing so good as the Fl. Ext. of cascara sagrada—Parke, Davis & Co.'s preparation—together with the occasional use of salts. In cases where epsom salts are objectional by the mouth on account of the nausea excited, they are very efficacious given in saturated solution, high up in the bowel, per rectum.

The other case I have to relate is connected with the other end of the intestinal tract.

CASE II.—A woman of twenty years of age, in her first pregnancy, was greatly nauseated during the first three months. During the next four months she lived in comparative comfort, but occasionally would suffer from attacks of nausea. During the last two months, however, about midnight she would awaken with great distress in her stomach, which would be finally relieved by vomiting a thick, glairy mucus. During the day time she had unpleasant feelings, excepting occasionally a sore throat without visible lesion; she was slightly constipated, and this was readily overcome by cascara sagrada; but the relief from constipation did not affect the midnight nausea. Morphine, combined with cerium oxalate, gave the best results but did not relieve entirely.

Final release came only with the birth of the child, and, strange to say, during her labor, which lasted twenty-six hours, she did not vomit or have any feeling of nausea except at the midnight hour.

I have no remarks to offer on this case, except that as it was a little out of the usual run, I thought it worth reporting.

Dr. A. E. Persons.—I have seen these disturbances, not so much during pregnancy, but under certain other conditions. Have seen large impactions of feces, which were diagnosticated as pus cavities, which it was proposed to open and wash out. A careful examination showed the tumor to be fecal in character, and an enema removed the mass. I believe that in the latter months of pregnancy impaction is due to the pressure on the intestines and the consequent innervation, producing a constriction at one end and a dilatation at the other. In cases of persistent vomiting and nausea, some abnormal condition must be present, and hence stomach sedatives had better give way to cervix sedatives.

Dr. E. Clark.—I have found Parke, Davis & Co.'s preparation of cascara sagrada one of the best remedies for impaction and constipation. Nux vomica relieves somewhat the torpor of the intestinal muscle. I think that fecal accumulations can go on, and yet the patient may have a daily evacuation of the bowels.

Dr. Geo. E. Fell.—I have had some good results with P., D. & Co.'s preparation of cascara, and have combined it with Wampole's cod liver oil. I believe it is advantageous to combine a tonic with a cathartic.

Dr. W. W. Potter.—The lesson to be drawn is that not much reliance is to be placed upon what the patient says about her evacuations. Impactions may become softened near the center, forming a sort of a tunnel, and daily evacuations may follow. Persistent nausea is a difficult question. Cocaine, given internally, has proven very efficient. But it becomes necessary in some cases to resort to abortion in order to save the patient's life. I have seen one patient die of inanition produced by persistent nausea, where abortion was proposed but declined.

Dr. Thos. Lothrop.—I have relied more upon colocynth co, hyoscyamus, and nux vomica, than cascara sagrada in the treatment of constipation of pregnancy, and have had excellent results. I do not believe that any one remedy can be relied upon in the vomiting of pregnancy. Cocaine will do nicely in one case, and will utterly fail in another, the reason being our inability to reach the cause.

Dr. R. L. Banta.—I would not give P., D. & Co.'s preparation ot cascara for personal reasons. We do not meet the persistent vomiting so much in the later stages as in the second and third months of pregnancy. Cocaine has done some wonderful things in my practice. Oxalate of cerium in thirty to forty grain doses, three times daily, has produced good results. I rely, however, upon cocaine.

Dr. EUGENE SMITH presented a report of the following case of cephalhematomata as occasioned by the age of the mother and the large size of the child :

On February 13th I visited Mrs. R——, and found she had been having labor pains for ten hours. She was a primipara, forty years old. The abdominal tumor was large, the pains hard, the "waters" had broken and were dribbling away from time to time. An examination revealed the cervix soft and dilatable, the os the size of a twenty-five cent piece, the presentation L. O. A. Eight hours later I found no change, except that the cervix was readily dilatable when spread by the fingers, but thicker and more boggy, the so-called anterior lip being much puffed and swollen. This condition of the cervix became more marked in the succeeding ten hours, when the head had fairly engaged in the pelvic cavity. The cervix could be pushed up above the head between pains, but it came down again with every pain.

On the morning of the 14th of February,[1] I found the patient was losing courage and the pains were losing strength, becoming shorter and more spasmodic. Applying the White's forceps, I delivered the head after a series of efforts, which, like real pains, advanced the head gradually. Pulling with one hand and pushing up the swollen cervix with the other, the cervix finally remained above the head. The mother made a good recovery.

The child weighed fourteen pounds, and after birth showed a slight mark of the forceps blade over the frontal eminences, the forceps having adapted themselves to the head before rotation had taken place. This was the more difficult to prevent because the head slipped above the pelvic brim upon trying to lock the forceps after their application.

Twenty-four hours after birth the baby first showed a slight ecchymosis over the frontal bone, which slowly spread and deepened until the eyes were closed and the whole frontal region was deeply ecclymosed. On the third day the child refused to nurse, and later Cheyne-Stokes' respiration heralded its death, which occurred on the morning of the fourth day.

A post-mortem was not secured, but compression of the brain from clot formation was believed to be the cause of death, although no paralysis in the extremities was noticed.

1 To relieve distension of the bladder, the catheter was used at midnight.

